# Interleukin-33 Receptor (ST2) Deficiency Improves the Outcome of *Staphylococcus aureus*-Induced Septic Arthritis

**DOI:** 10.3389/fimmu.2018.00962

**Published:** 2018-05-16

**Authors:** Larissa Staurengo-Ferrari, Silvia C. Trevelin, Victor Fattori, Daniele C. Nascimento, Kalil A. de Lima, Jacinta S. Pelayo, Florêncio Figueiredo, Rubia Casagrande, Sandra Y. Fukada, Mauro M. Teixeira, Thiago M. Cunha, Foo Y. Liew, Rene D. Oliveira, Paulo Louzada-Junior, Fernando Q. Cunha, José C. Alves-Filho, Waldiceu A. Verri

**Affiliations:** ^1^Departamento de Patologia, Centro de Ciências Biológicas, Universidade Estadual de Londrina, Londrina, Brazil; ^2^Cardiovascular Division, British Heart Foundation Centre, King’s College London, London, United Kingdom; ^3^Department of Pharmacology, Ribeirão Preto Medical School, University of São Paulo, Ribeirão Preto, Brazil; ^4^Departamento de Microbiologia, Centro de Ciências Biológicas, Universidade Estadual de Londrina, Londrina, Brazil; ^5^Laboratory of Pathology, Faculty of Medicine, University of Brasilia, Brasilia, Brazil; ^6^Department of Pharmaceutical Sciences, Healthy Sciences Centre, Londrina State University, Londrina, Brazil; ^7^Department of Physics and Chemistry, School of Pharmaceutical Sciences of Ribeirão Preto, University of São Paulo, Ribeirão Preto, Brazil; ^8^Laboratório de Imunofarmacologia, Departamento de Bioquímica e Imunologia, Instituto de Ciencias Biologicas (ICB), Universidade Federal de Minas Gerais, Belo Horizonte, Brazil; ^9^Division of Immunology, Infection and Inflammation, University of Glasgow, Glasgow, United Kingdom; ^10^Division of Clinical Immunology, School of Medicine of Ribeirao Preto, University of São Paulo, Ribeirao Preto, Brazil

**Keywords:** interleukin-33, ST2, septic arthritis, *Staphylococcus aureus*, interferon-γ, nitric oxide, Th1, M1 macrophage

## Abstract

The ST2 receptor is a member of the Toll/IL-1R superfamily and interleukin-33 (IL-33) is its agonist. Recently, it has been demonstrated that IL-33/ST2 axis plays key roles in inflammation and immune mediated diseases. Here, we investigated the effect of ST2 deficiency in *Staphylococcus aureus*-induced septic arthritis physiopathology. Synovial fluid samples from septic arthritis and osteoarthritis individuals were assessed regarding IL-33 and soluble (s) ST2 levels. The IL-33 levels in samples from synovial fluid were significantly increased, whereas no sST2 levels were detected in patients with septic arthritis when compared with osteoarthritis individuals. The intra-articular injection of 1 × 10^7^ colony-forming unity/10 μl of *S. aureus* American Type Culture Collection 6538 in wild-type (WT) mice induced IL-33 and sST2 production with a profile resembling the observation in the synovial fluid of septic arthritis patients. Data using WT, and ST2 deficient (^−/−^) and interferon-γ (IFN-γ)^−/−^ mice showed that ST2 deficiency shifts the immune balance toward a type 1 immune response that contributes to eliminating the infection due to enhanced microbicide effect *via* NO production by neutrophils and macrophages. In fact, the treatment of ST2^−/−^ bone marrow-derived macrophage cells with anti-IFN-γ abrogates the beneficial phenotype in the absence of ST2, which confirms that ST2 deficiency leads to IFN-γ expression and boosts the bacterial killing activity of macrophages against *S. aureus*. In agreement, WT cells achieved similar immune response to ST2 deficiency by IFN-γ treatment. The present results unveil a previously unrecognized beneficial effect of ST2 deficiency in *S. aureus*-induced septic arthritis.

## Introduction

Interleukin-33 (IL-33) is a member of the IL-1 cytokine family that can act either as a chromatin-associated nuclear factor or as a classic cytokine (1, 2). Once released, IL-33 binds to the heterodimeric receptor complex consisting of ST2 and IL-1 receptor accessory protein recruiting typical intracellular proteins of the toll-like receptor (TLR)/IL-1 superfamily (3, 4). The transmembrane form of ST2, encoded by the *ST2* gene is expressed by cells including activated Th2 cells (5), mast cells (6), and ILC2 (7). ST2 is alternatively spliced to produce a soluble form (sST2), which acts as an IL-33 scavenger (8). Antibodies targeting ST2, ST2-Fc fusion proteins or ST2 deficient mice contributed to demonstrate that the lack of IL-33/ST2 signaling favors the expansion of Th1 cells and inhibits Th2 cell-mediated immune responses (5, 8–11).

Conversely, IL-33/ST2 signaling has now emerging pleiotropic properties, including type 1 and 3 immunity and regulatory patterns (4, 12). Indeed, IL-33/ST2 has a proinflammatory role in Th1 and Th17 immune responses (13, 14). Both, IL-33 and ST2 are expressed in the human and mouse model of rheumatoid arthritis (RA) synovial tissue, are elevated in the sera and synovial fluids of RA patients and manifest correlation with disease progression (14–18). Endothelial cells and fibroblasts constitutively express high levels of IL-33 mRNA and protein, indicating that they are a key source of IL-33 in the inflamed synovium (11, 16). Thus, IL-33 and ST2 are expressed by joint cells in inflammatory conditions.

During infections, IL-33/ST2 signaling plays dual roles depending on the organ involved and the Th1/Th2 shifting necessary to better control the infectious foci (4). IL-33 is protective during acute phase of sepsis (19), keratitis caused by *Pseudomonas aeruginosa* (20) or *Staphylococcus aureus* wound infection (21) and in parasitic diseases with *Trichuris muris* (22), *Schistosoma mansoni* (23), or *Toxoplasma gondii* (24), whereas it is deleterious during cutaneous and visceral leishmaniasis (10, 25, 26). Thus, strategies targeting IL-33/ST2 pathway should account the cytokine milieu and disease context.

Septic arthritis is considered as one of the most aggressive joint diseases due to its rapidly progressive disease profile, pain, severe joint lesion, and dysfunction even with therapy onset (27, 28). Patients with underlying joint diseases, such as RA are 4- to 15-fold more susceptible to septic arthritis than general population (29). Joint lesions facilitate bacterial colonization together with a reduced immune response due to chronic treatment with disease modifying drugs, corticosteroids and biologic therapies that cause patient immune suppression (30–32). *S. aureus* is the most common cause of SA (31). There is evidence that both drug resistant *S. aureus* such as methicillin-resistant *S. aureus* and non-drug resistant *S. aureus* cause septic arthritis (28, 29, 33, 34). The fast development of joint destruction in septic arthritis supports the urgent need for development of new treatment strategies against *S. aureus* arthritis.

As IL-33 and its receptor ST2 have been recognized as an important axis in joint inflammation and infectious diseases, we therefore, investigated whether ST2 deficiency would influence the outcome and contributing mechanisms of this receptor in *S. aureus*-induced septic arthritis.

## Materials and Methods

### Animals

Male BALB/c [wild-type (WT)], ST2^−/−^ (BALB/c background), C57BL/6 (WT), and interferon-γ (IFN-γ)^−/−^ (C57BL/6 background) mice were used in this study. ST2^−/−^ mice were originally obtained from Dr. Andrew McKenzie (LMB, Cambridge) (15). IFN-γ^−/−^ mice were obtained from Jackson Laboratories (Bar Harbor, ME, USA).

A total of 1,360 mice were used in this study. All mice were housed in standard clear plastic cages with free access to water and food, and temperature of 23°C ± 2 at constant humidity. A 12/12 h light/dark cycle was used with lights on at 6 a.m. and off at 6 p.m. The behavioral tests were performed between 9 a.m. and 5 p.m. in a temperature-controlled room (23°C ± 2). Animal care and handling procedures were in accordance with the International Association for Study of Pain guidelines, and all protocols were approved by the Ethics Committee of the Londrina State University (OF.CIRC.CEUA, process number 20165/2009).

### Clinical Samples

Synovial fluid samples from 4 to 5 individuals with septic arthritis and 10 osteoarthritis individuals were collected in order to assess IL-33, sST2, and IFN-γ levels using ELISA kits (R&D Systems, Minneapolis, MN, USA). All individuals were recruited at the Division of Rheumatology, Hospital das Clínicas, Ribeirão Preto Medical School (HC-FMRP), São Paulo, Brazil, and were informed about the aims of the study and provided written consent before participating. The Human Ethics Committee of the FMRP approved this study (Process number 4971/2012).

### Mouse Model of *S. aureus*-Induced Arthritis

*Staphylococcus aureus* was obtained from American Type Culture Collection (ATCC, USA) number 6538. Septic arthritis was induced by local injection of 10^7^ colony-forming unity (CFU) of *S. aureus* in 10 µl in sterile PBS into the right knee joints. Intra-articular (i.a.) injection of 10 µl of sterile saline was used as negative control group. To assess the intensity of arthritis, a clinical score was carried out using macroscopic inspection of the knee joints yielding a score of 0–4 for each limb (0—normal, 1—periarticular erythema, 2—articular erythema and edema, 3—function loss with difficult locomotion and articular extension, 4—purulent process with abscess formation) (28).

### Assessment of Articular Hyperalgesia

Articular mechanical hyperalgesia was assessed over 27 days post-i.a. infection with *S. aureus* using an electronic pressure meter (IITC 152 Inc., Life Science Instruments California, CA, USA) (35). The electronic pressure-meter apparatus automatically recorded the intensity of the force applied when the paw was withdrawn. The results were expressed as the flexion-elicited withdrawal threshold in grams.

### Determination of Knee Joint Edema

Knee joint edema was assessed over 27 days post-i.a. infection with *S. aureus* using a digital caliper (Digmatic Caliper, Mitutoyo Corp., Kanagawa, Japan). The results were expressed as the difference (delta, Δ) between the diameter measured before (basal) and after induction of articular infection in millimeter.

### Quantification of Cytokines

Knee joints were dissected out and frozen with liquid nitrogen. Then, samples were homogenized in a buffer containing a cocktail of protease inhibitors [NaCl 0.4 M, Tween 20 0.05%, bovine albumin 0.5%, phenyl methyl sulphonyl fluoride 0.1 mM, benzethonium chloride 0.1 mM, EDTA 10 mM, aprotinin 20 KI·ml^−1^ (0.01 mg·ml^−1^) diluted in phosphate buffer saline pH 7.4], centrifuged and the supernatants were used to determine the levels of sST2, IL-33, TNF-α, IL-1β, IFN-γ, IL-4, IL-5, IL-17, and IL-10. The results were expressed as picogram per 100 mg of tissue (36). Supernatants from bone marrow-derived macrophages (BMDMs) culture were also collected to determine the levels of IL-33 and IFN-γ and the results were expressed as picogram/milliliter. All measurements were performed using ELISA kits from R&D Systems (Minneapolis, MN, USA) or eBioscience (San Siego, CA, USA). The minimum sensitivity of the kits was ≥0.7 pg/ml.

### Determination of Joint Leukocyte Infiltration and Bacterial Counts

Knee joints cavities were exposed and washed with the aid of a pipette three times with a total volume of 10 µl of sterile saline plus 1 mM EDTA. Each washing procedure used approximately 3.3 µl of saline. The total number of leukocytes was determined in a Neubauer chamber diluted in Turk’s solution and differential cell counts were performed in Rosenfeld stained slices using a light microscope. The results were expressed as the number of total of leukocytes, neutrophils, or mononuclear cells × 10^4^ (mean ± SEM)/per cavity. The same samples were plated on blood agar to determine the bacterial load in the joints and the results were expressed as CFU per cavity. Additionally, spleen from the same animals were explanted and plated on blood agar to determine the bacterial load. The results were expressed as CFU per spleen.

### Histological Analysis

Whole knee joints were removed and fixed in 4% formaldehyde for 2 days before decalcification in 5% formic acid and processing for paraffin embedding. Tissue sections (5 µm) were stained with hematoxylin and eosin. All the slides were coded and assessed in a blinded manner by two observers regarding the degree of synovitis (leukocyte infiltration score) and cartilage destruction.

### Proteoglycan Quantification Assay

Chondroitin sulfate from patella samples was quantitated using 1,9-dimethyl-methylene blue assay. The glycosaminoglycan content of samples was calculated from the standard curve of chondroitin sulfate (14).

### RT-qPCR

Total RNA was extracted from 1 × 10^6^ *S. aureus*-infected BMDMs [multiplicity of infection (MOI): 3] and from whole knee joint samples using TRIzol reagent (Invitrogen). For RT-qPCR, the total RNA was extracted with SV Total RNA Isolation System Kit (Promega, USA) according to the manufacturer’s instructions. RT-PCR and qPCR were performed using GoTaq^®^ 2-Step RT-qPCR System (Promega) on a StepOnePlus™Real-Time PCR System (Applied Biosystems^®^) using primers for *Rankl, Rank, Opg, IFN-γ, iNOS, IL-33*, and *ST2*. Raw data were normalized to *Gadph* expression and were analyzed by the 2^−(ΔΔ^*^Ct^*^)^ method.

### Western Blot

A total of 2 × 10^6^ BMDMs were seeded per well and pretreated with IFN-γ (100 U/ml, Invitrogen), anti-IFN-γ (10 µg/ml, R&D Systems), or medium for 1 h followed by infection with *S. aureus* (MOI = 3) for 18 h. The supernatants were collected to further assess the nitrite production. The cell lysates were collected using RIPA buffer containing CST—Protease/Phosphatase Inhibitor Cocktail (Cell Signaling, USA). Whole knee joint samples using RIPA buffer containing Protease/Phosphatase Inhibitor Cocktail (Cell Signaling, USA) were also collected. Total protein from cells and knee joint samples were quantified and the lysates were mixed with 4× Laemmli sample buffer (Sigma-Aldrich). Antibody against iNOS (1:10,000, Sigma Chemical Co., St. Louis, MO, USA) was used for protein detection after electrophoresis in 10% SDS-PAGE gel, transference into nitrocellulose membrane (Merck Millipore, USA), and blocking. HRP-conjugated secondary antibody was used (KPL, USA). Immunodetection was performed using an enhanced chemiluminescence technique (ChemiDoc XRS System, Biorad Laboratories). The membrane was stripped and reprobed with β-actin (1:5,000, Sigma Chemical Co., St. Louis, MO, USA) as a loading control. Densitometry data were measured after normalization to the control (house-keeping gene, β-actin) using Scientific Imaging Systems (Image Lab 3.0 software; Biorad Laboratories, Hercules, CA, USA). Full scan of the original uncropped western blot is shown in Figure S4 in Supplementary Material.

### FITC-Labeling of Staphylococci

*Staphylococcus aureus* ATCC 6538 was grown to mid-log phase in fresh Muller–Hinton broth. Bacteria were washed twice with sterile PBS and labeled in 0.1 mg/ml FITC (Sigma Chemical Co., St. Louis, MO, USA) for 1 h at 37°C with shaking. Prior to use, bacteria were washed twice with PBS and re-suspended in Hank’s solution (Sigma Chemical Co., St. Louis, MO, USA).

### Flow Cytometry Analysis

Phagocytosis assay and detection of intracellular cytokines was performed using an FACS Verse (BD Biosciences, San Diego, CA, USA) flow cytometer. Phagocytosis of naïve BMDMs and naïve neutrophils was measured using FITC-labeled *S. aureus* as previously described in Ref. (37). In other set of experiments to assess the intracellular cytokines, draining popliteal lymph nodes (LNs) were collected from naïve and infected mice at indicated times post-infection and processed as a pool. Cell suspensions obtained (1 × 10^6^ cells) were stained with fluorochrome-conjugated antibodies for CD4 (H129.19), IFN-γ (B27), IL-4 (11B11), IL-17 (TC11-18H10), or IL-10 (JES5-16E3) from BD Biosciences (San Diego, CA, USA). Data were analyzed with FlowJo software (TreeStar, Ashland, OR, USA).

### Killing Assay

To obtain macrophages (BMDM), cells were differentiated during 6 days in RPMI medium plus 20% supernatant from L929 cells (38). To obtain neutrophils, cells were isolated by Percoll gradient (39). BMDM or neutrophils (1 × 10^6^/well) were then pretreated with IFN-γ (100 U/ml), anti-IFN-γ (10 µg/ml), or medium for 1 h followed by infection with *S. aureus* (MOI = 3). Other wells with only bacteria and RPMI medium were prepared as a positive control. The plates were centrifuged for 3 min at 5,000 rpm and returned to the cell incubator for 3 h. The supernatants were collected to further assess the nitrite production. Cells were then lysed by addition of Triton X-100 0.2%. The lysates were serially diluted 1:100,000 in 1 × PBS and plated on blood agar plates and incubated overnight at 37°C. The results were expressed as % of viable bacteria by comparing with the positive control.

### Nitrite Determination

Nitrite (NO_2_^−^) accumulation, as an indication of NO production was measured using Griess reagent. In this assay, 0.1 ml of sample was mixed with 0.1 ml of Griess reagent in a multiwell plate, and the absorbance was read at 550 nm 10 min later. Nitrite concentrations were determined by reference to a standard curve of sodium nitrite (1–200 µmol/l) (40).

### Pharmacological Treatment of Mice

In some experiments, infected WT and ST2^−/−^ were treated intraperitoneally with 30 mg/kg of aminoguanidine (AMG; Sigma, Chemical Co., St. Louis, MO, USA), a selective iNOS inhibitor, for 28 consecutive days (once a day). Mice were euthanized at the 28th day post-infection, and inflammatory parameters and bacterial load in joint and spleen tissues were analyzed.

### Culture of Macrophages Like-Cells

Peritoneal cells from naïve and infected mice were cultured in RPMI medium for 4 h to allow macrophages to adhere. The floating cells were washed away and the adherent macrophages were challenged with lipoteichoic acid (LTA, 10 µg/ml), a TLR2 agonist plus IFN-γ (100 U/ml) in RPMI for 24 h at 37°C. The supernatants were harvested to assess the NO_2_^−^ accumulation.

### Statistical Analysis

Statistical significance was analyzed using PRISM 6.01 (GraphPad Software, USA). The data are expressed as the mean ± SEM. Statistical differences were considered when *P* < 0.05.

## Results

### IL-33 and sST2 Levels in Synovial Fluids of Septic Arthritis and Osteoarthritis Patients, and a Similar Profile in Mouse Septic Arthritis

**Figure 1:**
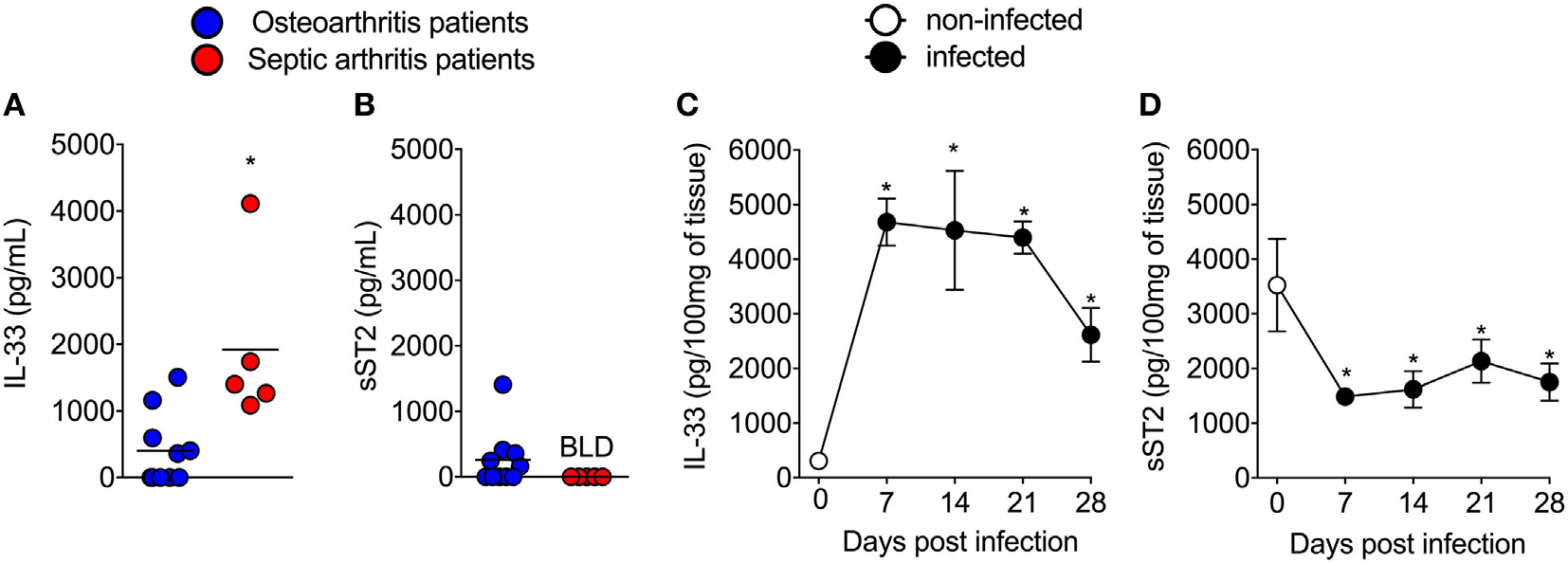
Interleukin-33 (IL-33) and sST2 levels in synovial fluid samples of septic arthritis and osteoarthritis patients, and similar profile in mouse septic arthritis. **(A,B)** Synovial fluid samples from patients with septic arthritis and osteoarthritis were collected and processed to determine the levels of IL-33 and sST2 by ELISA. **(C,D)** *Staphylococcus aureus* or saline (day 0) was injected in in the femur-tibial joint of wild-type and knee joints samples were collected and processed to determine the levels of IL-33 and sST2 at indicated points (7–28 days) post *S. aureus* injection by ELISA. For clinical samples analysis: *n* = 5 for septic arthritis and *n* = 10 for osteoarthritis patients. *P < 0.05 vs osteoarthritic patients group **(A,B)**. Kruskal–Wallis test followed by Dunn’s test. For mice samples analysis: *n* = 6 per group, representative of two independent experiments. *P < 0.05 vs day 0 of infection **(C,D)**. Two-tailed unpaired Student’s *t*-test.

First, the IL-33 and sST2 levels in the synovial fluid of patients with septic arthritis and osteoarthritis were determined. Clinical and demographic characteristics of these patients are presented as Table S1 in Supplementary Material. IL-33 levels were higher in the synovial fluid of SA patients than in the synovial fluid OA patients (Figure 1A). The levels of sST2 were below limit detection in septic arthritis patients (Figure 1B). These clinical results indicate that there are higher levels of IL-33 in septic arthritis primary foci than in osteoarthritis. In mice, the i.a. injection of *S. aureus* induced the increase of IL-33 levels (Figure 1C) and decreased of sST2 levels (Figure 1D) in the knee joints that lasted for 28 days compared with day 0 (representative group that received only saline). Despite the detection of IL-33 in the synovial fluid and knee joints of septic arthritis patients and mice, respectively, whether IL-33 and its receptor ST2 have a function in disease is unknown.

### ST2 Receptor Deficiency Ameliorates *S. aureus*-Induced Septic Arthritis

**Figure 2:**
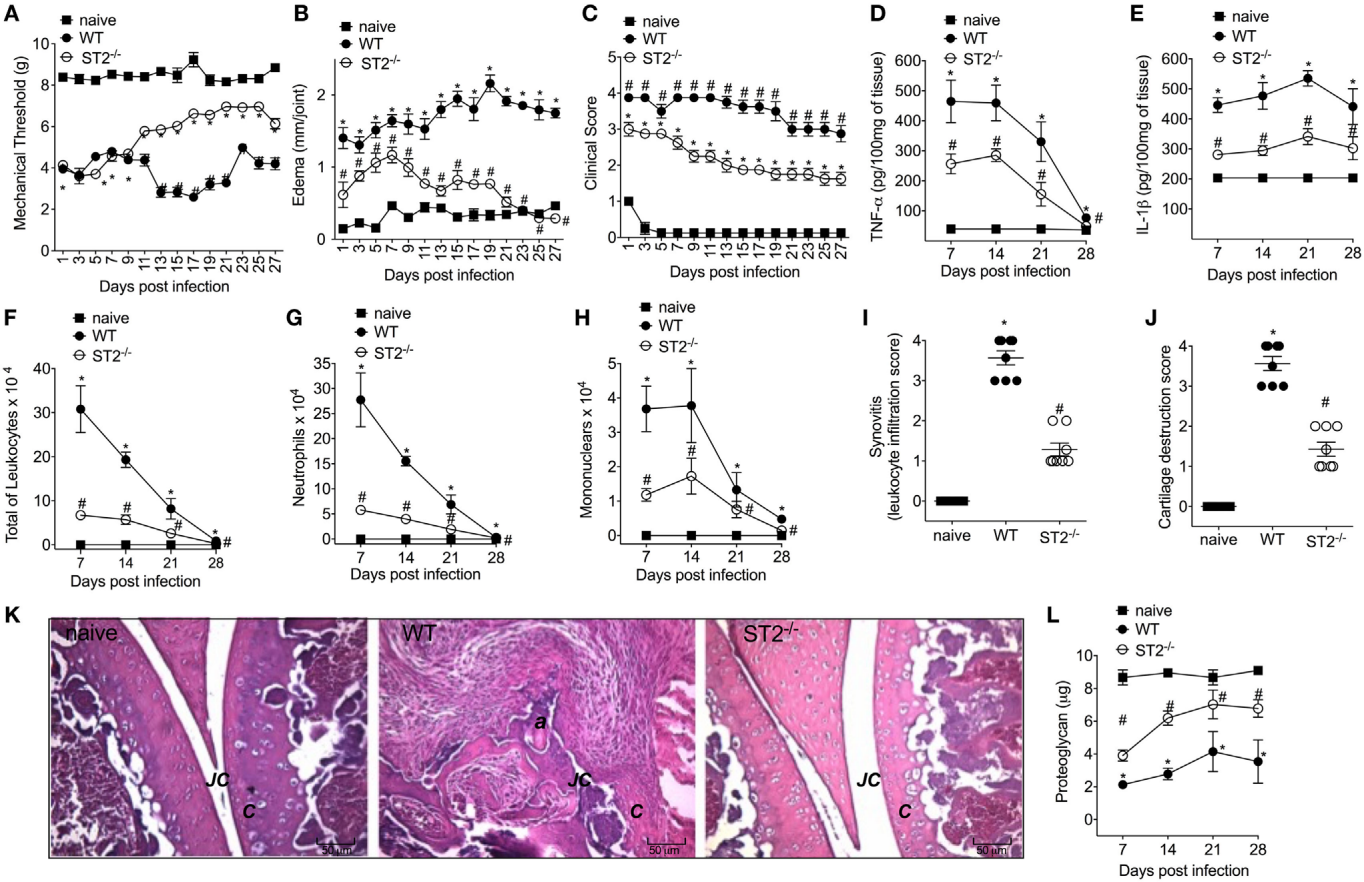
ST2 deficiency ameliorates *Staphylococcus aureus*-induced septic arthritis. *S. aureus* or saline was injected in the femur-tibial joint of wild-type (WT) and ST2^−/−^ mice. **(A)** Mechanical hyperalgesia, **(B)** articular edema, and **(C)** clinical score were evaluated over 27 days post-infection. Knee joints were collected and processed to determine the levels of **(D)** TNF-α and **(E)** IL-1β by ELISA determined at days 7–28 days post-infection. **(F)** Total leukocytes, **(G)** neutrophil, and **(H)** mononuclear recruitment to the knee joint were determined at 7–28 days post-infection. Knee joint samples were collected at the 28th day post-infection for histological analysis by hematoxylin/eosin stained slices to determine: **(I)** synovitis score (intensity: 1–4) and **(J)** cartilage destruction score (intensity: 1–4). **(K)** Representative images of knee joints at 28 post-infection in original magnification ×10. The letter ***a*** indicates a heavily inflamed joint with cartilage destruction and pannus formation. **(F)** Proteoglycan content in patella determined at 7–28 days post-infection. For inflammatory parameters and proteoglycan content: *n* = 6 per group per *in vivo* experiment, representative of two independent experiments. *P < 0.05 vs naïve mice group, ^#^*P* < 0.05 vs WT mice group **(A–H,L)**. One-way ANOVA followed by Tukey’s test. For histological analysis: *n* = 8 per group per experiment, representative of two independent experiments. *P < 0.05 vs naïve mice group, ^#^*P* < 0.05 vs WT mice group **(I–K)**. Kruskal–Wallis test followed by Dunn’s test. Abbreviations: ***C***, cartilage; ***JC***, joint cavity.

Considering that septic arthritis triggered the production of IL-33 and reduction of sST2 levels in the synovial fluid of patients (Figure 1A), we used a model of *S. aureus*-induced septic arthritis in WT (balb/c) and ST2^−/−^ mice to investigate the disease outcome in the ST2 deficiency scenario. i.a. injection of *S. aureus* increased mechanical hyperalgesia in WT mice as observed by reduction in the mechanical threshold when compared with naïve mice (Figure 2A). Interestingly, the *S. aureus*-induced hyperalgesia was similar between WT and ST2^−/−^ mice up to 9 days of infection. However, from 11 days onward, *S. aureus*-induced hyperalgesia started to reduce in ST2^−/−^ compared with WT mice. This result may have, at least, two explanations; ST2^−/−^ mice respond better than WT against *S. aureus* infection or by lacking ST2, these mice would present a reduction of hyperalgesia since IL-33 is a hyperalgesic cytokine (13). Moreover, IL-33 also mediates the paw edema induced by carrageenan (41). Indeed, WT mice presented increased edema when compared with ST2^−/−^ mice (Figure 2B). The reduction of hyperalgesia and edema in ST2^−/−^ mice was followed by lower clinical score as well (Figure 2C). WT mice also showed increased levels of TNF-α and IL-1β after infection (Figures 2D,E, respectively), inflammatory cell recruitment to the knee joint (Figures 2F–H), and tissue inflammatory cell infiltration demonstrated histologically (Figures 2I,K) when compared with ST2^−/−^ mice. Ultimately, these features resulted in increased cartilage destruction with only parts of cartilage remaining (Figures 2J,K), which was confirmed by proteoglycan content loss (Figure 2L). These morphological changes were reduced in ST2^−/−^ mice (Figures 2I–K). Further, WT mice presented increased mRNA expression of receptor activator of nuclear factor-kappa B ligand (Figure S1A in Supplementary Material) and receptor activator of nuclear factor-kappa B (Figure S1B in Supplementary Material), and reduced mRNA expression of OPG (osteoprotegerin, Figure S1C in Supplementary Material), an expression pattern indicating bone resorption (42). These results indicate that IL-33/ST2 signaling deficiency ameliorated the clinical features of septic arthritis. Therefore, we next examined whether the reduction of disease intensity in ST2^−/−^ mice would be related to reducing inflammation since IL-33/ST2 signaling triggers inflammatory responses, or if ST2 deficiency would enhance the anti-microbial response due to boosting the immune response pattern necessary to combat *S. aureus* infection.

### ST2 Deficiency Enhances Neutrophil and Macrophages Bactericidal Activity Against *S. aureus*

**Figure 3:**
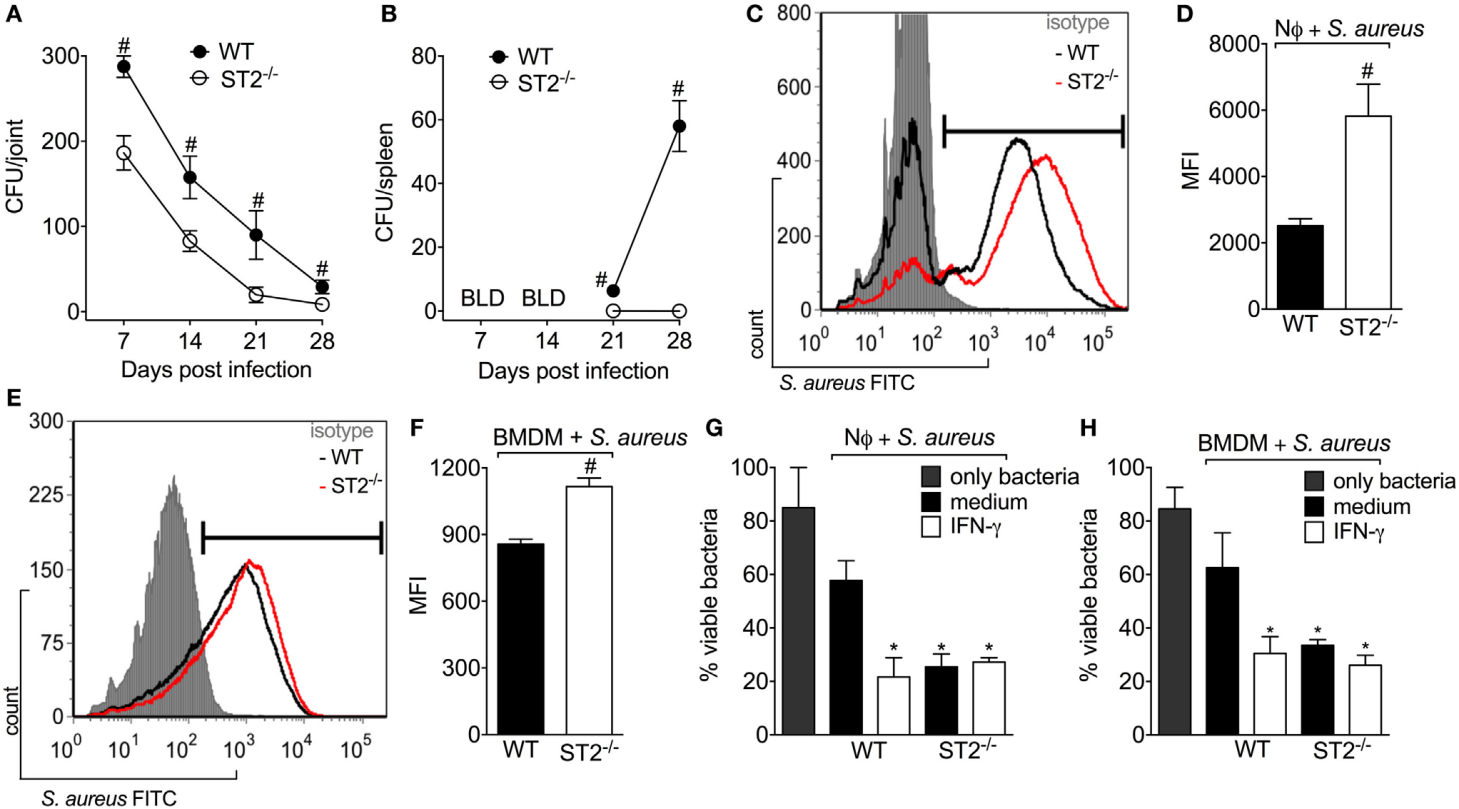
ST2 deficiency enhances neutrophil and macrophages bactericidal activity against *Staphylococcus aureus. S. aureus* was injected in the femur-tibial joint of wild-type (WT) and ST2^−/−^ mice. At indicated points (7–28 days post-infection), **(A)** knee joints and **(B)** spleen samples were collected and bacterial counts were determined on agar dishes. **(C,D)** FACS analysis of neutrophils (1 × 10^6^) from WT and ST2^−/−^ naïve mice incubated *in vitro* with *S. aureus* at a multiplicity of infection (MOI) of 3 to evaluate phagocytosis. **(E,F)** FACS analysis of naïve bone marrow-derived macrophages (BMDMs) (1 × 10^6^) from WT and ST2^−/−^ naïve mice incubated *in vitro* with *S. aureus* at a MOI of 3 to evaluate phagocytosis. Microbicidal activity of neutrophils **(G)** and BMDM **(H)** from WT and ST2^−/−^ naïve mice preincubated with interferon-γ (IFN-γ) (100 IU/ml, 1 h) against *S. aureus*. All neutrophils were harvested from the bone marrow of mice. *N* = 6 wells per group per *in vitro* experiment, representative of two independent experiments. One-way ANOVA followed by Tukey’s test. ^#^*P* < 0.05 vs WT mice group **(A,B)**. Samples were pooled from 10 mice per group per *in vitro* experiment, representative of two independent experiments. One-way ANOVA followed by Tukey’s test. ^#^*P* < 0.05 vs WT neutrophils or BMDM group **(C–F)**. *P < 0.05 vs WT group preincubated with only medium **(G,H)**.

Inhibiting IL-33/ST2 signaling reduces inflammation induced by carrageenan and LPS, and in RA by targeting neutrophilic influx (14, 41, 43, 44). On the other hand, neutrophils have a fundamental role in controlling the bacterial load in infections. Indeed, the treatment with IL-33 improves the sepsis outcome due to inhibition of LPS-induced CXCR2 internalization, which maintains the neutrophil migration toward the infectious foci (19). To ascertain this issue and considering that mice lacking ST2 showed a protective phenotype against *S. aureus* local inflammation in septic arthritis (Figure 2), first, we evaluated if there was any difference in the bacterial load in WT and ST2^−/−^ mice. Bacterial recovery showed that WT mice presented detectable *S. aureus* CFU in both the knee joint and the spleen (Figures 3A,B, respectively). On the other hand, ST2^−/−^ mice presented reduced CFU number compared with WT mice as well as did not present *S. aureus* in the spleen, which indicates that the infection remained local in ST2^−/−^, but not in WT mice (Figure 3B). Given neutrophils and macrophages are the initial defenders against *S. aureus* infection (45), we determined if ST2 deficiency would influence neutrophil and macrophage phagocytosis and killing of *S. aureus*. Using FITC-labeled *S. aureus*, we found that both neutrophils (Figures 3C,D) and macrophages (BMDMs) (Figures 3E,F) from WT mice presented reduced phagocytic capacity when compared with ST2^−/−^ cells. In addition, neutrophils and BMDMs from WT mice also partially controlled bacterial growth, and, importantly, neutrophils and BMDMs from WT mice increased their bacterial killing after stimulation with recombinant IFN-γ (Figures 3G,H) in a manner that reached the capability of ST2^−/−^ cells. In contrast, the IFN-γ treatment of neutrophils and BMDMs from ST2^−/−^ mice did not further enhanced their bactericidal effect (Figures 3G,H), possibly because this pathway was already being enhanced by ST2 deficiency. Thus, these results show that impaired endogenous IL-33/ST2 signaling enhances neutrophil and macrophage bacterial killing with a profile that can be matched by IFN-γ treatment. Therefore, IFN-γ-related mechanisms were investigated in the following experiments (46).

### ST2 Deficiency Enhances NO Production by Neutrophils and Macrophages and Reduces *S. aureus*-Induced Septic Arthritis

**Figure 4:**
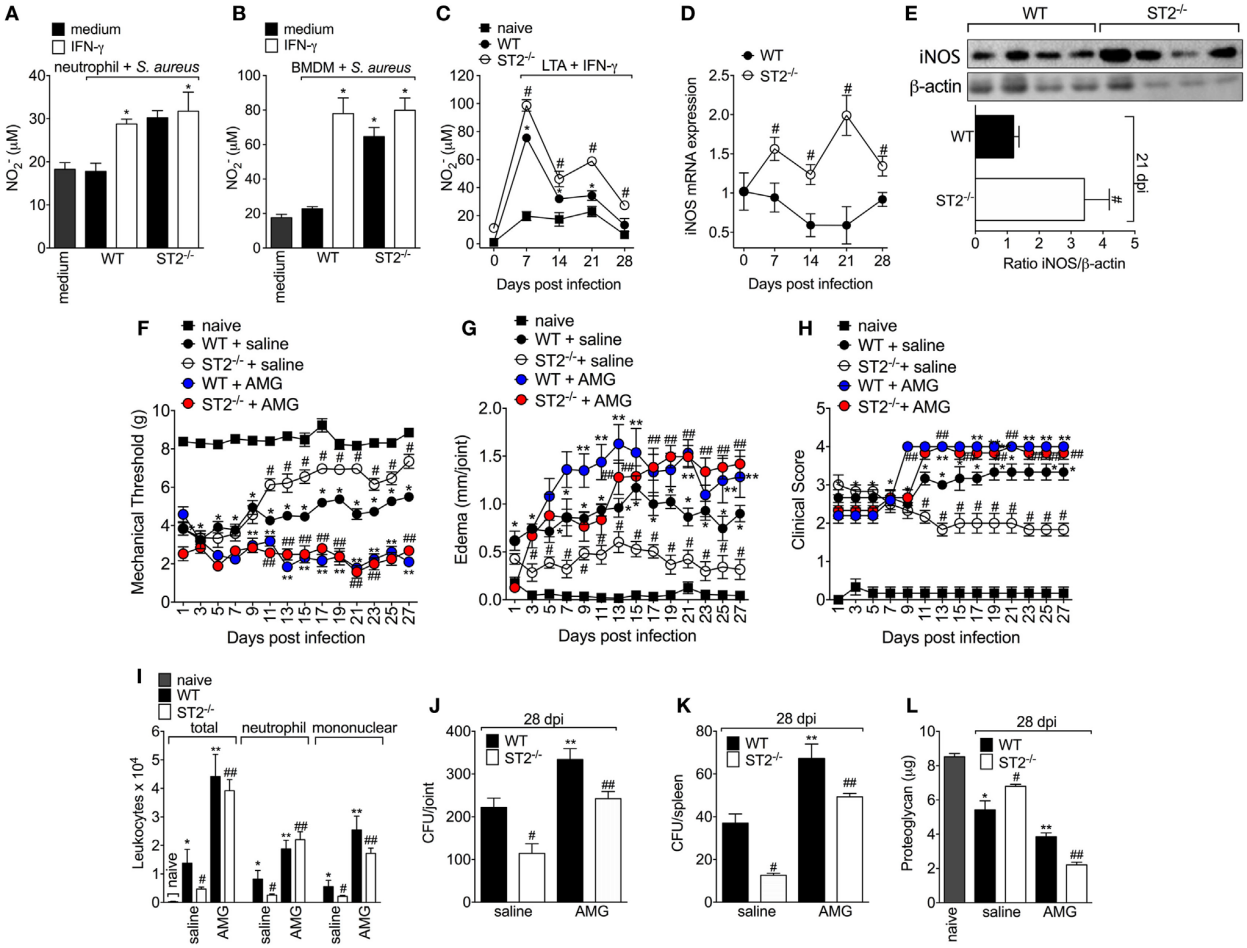
ST2 deficiency enhances NO production by neutrophils and macrophages and reduces *Staphylococcus aureus*-induced septic arthritis. NO production was determined as nitrite concentration by Griess reagent in the culture supernatant of **(A)** neutrophils and **(B)** bone marrow-derived macrophages (BMDMs) cells from wild-type (WT) or ST2 ^−/−^ naïve mice preincubated *in vitro* with interferon-γ (IFN-γ) (100 IU/ml, 1 h) or medium, followed by incubation with *S. aureus*, or **(C)** in the culture supernatant of macrophages like-cells isolated from peritoneal cavity of WT or ST2^−/−^ mice with staphylococcal arthritis and challenged with lipoteichoic acid (LTA) (10 µg/ml, a toll-like receptor 2 agonist) plus IFN-γ (100 UI/ml) for 48 h. *S. aureus* or saline (day 0) was injected in the femur-tibial joint of WT and ST2^−/−^ mice and knee joint samples were collected and processed to determine: **(D)** the mRNA and **(E)** protein expression of iNOS at indicated time points post-infection by qPCR and Western Blot, respectively. WT and ST2^−/−^ mice were treated with aminoguanidine (AMG, 30 mg/kg, s.c., 150 µl) or vehicle (saline, 150 µl) over 28 days after i.a. *S. aureus* [10^7^ colony-forming unity (CFU)/10 μl/joint] injection: **(F)** mechanical hyperalgesia, **(G)** articular edema, and **(H)** clinical severity score were evaluated over 27 days post-bacterial infection. At the 28th day post-infection, **(I)** leukocyte recruitment to the articular cavity, **(J)** bacterial counts in knee joint cavity and **(K)** spleen, and **(L)** proteoglycan content in patella samples were determined. *N* = 6 per group per *in vivo* experiment or *N* = 4 per group for WB analysis and samples were pooled from 10 mice per *in vitro* experiment. *P < 0.05 vs WT neutrophils or BMDM group preincubated with only medium, or vs mice naïve group; ^#^*P* < 0.05 vs WT and ST2^−/−^ neutrophils or BMDM group preincubated with only medium, or vs WT mice group **(A–E)**. *P < 0.05 vs naïve mice group; ^#^*P* < 0.05 vs ST2^−/−^ + saline mice group vs WT + saline mice group; **P < 0.05 WT + AMG mice group vs WT + saline mice group; ^##^*P* < 0.05 ST2^−/−^ + AMG mice group vs ST2^−/−^ + saline mice group **(F–L)**. Representative of two independent experiments. One-way ANOVA followed by Tukey’s test.

Since one of the IFN-γ mechanisms of bacterial killing is by increasing NO production in an iNOS-dependent manner (47) and that NO can also drive type 1 response (48–50), it was evaluated whether the protective phenotype of ST2 deficiency was related to an increase of IFN-γ/iNOS/NO signaling. First, it was measured the NO_2_^−^ levels in macrophages and neutrophils, which is indicative of iNOS activity. We found that neutrophils from WT mice presented lower production of NO_2_^−^ than neutrophils from ST2^−/−^ mice (Figure 4A) as well as IFN-γ enhanced the NO_2_^−^ production by WT neutrophils, but not ST2^−/−^ neutrophils. In accordance, naïve macrophages (BMDMs) infected with *S. aureus* or peritoneal macrophages collected at 7–28 days post *S. aureus* joint infection from WT presented reduced levels of NO_2_^−^ when compared with ST2^−/−^ cells (Figures 4B,C, respectively). Considering neutrophils and macrophages from WT mice presented reduced NO_2_^−^ levels compared with ST2^−/−^ cells, the iNOS expression was investigated in knee joint samples from WT and ST2^−/−^ mice with staphylococcal arthritis. Corroborating, both iNOS mRNA expression and protein (day 21, peak of mRNA expression) were reduced in the knee joints from WT mice compared with ST2^−/−^ mice (Figures 4D,E, respectively). Thus, to determine the contribution of iNOS to IL-33/ST2 signaling role in septic arthritis, WT and ST2^−/−^mice with septic arthritis received daily treatment with AMG, a selective iNOS inhibitor. Treatment with AMG reverted the protective effect of ST2 deficiency and also worsened the phenotype of WT mice, as observed by increased hyperalgesia (reduced mechanical threshold, Figure 4F), edema (Figure 4G), and clinical score (Figure 4H), when compared with the group that receive only vehicle. Similarly, the treatment with AMG in ST2^−/−^ mice increased leukocyte recruitment to the knee joint (Figure 4I), CFU count in both knee joint (Figure 4J) and spleen (Figure 4K), and proteoglycan degradation (Figure 4L) at the 28th day post-infection (Figures 4I–L). Altogether, these data indicate that ST2 receptor deficiency ameliorates *S. aureus*-induced septic arthritis by enhancing iNOS expression and thereby increasing NO levels, which is an important bactericidal mechanism and inductor of type 1 response.

### ST2 Deficiency Enhances Type 1-Driven Immune Response Against *S. aureus* in Septic Arthritis

**Figure 5:**
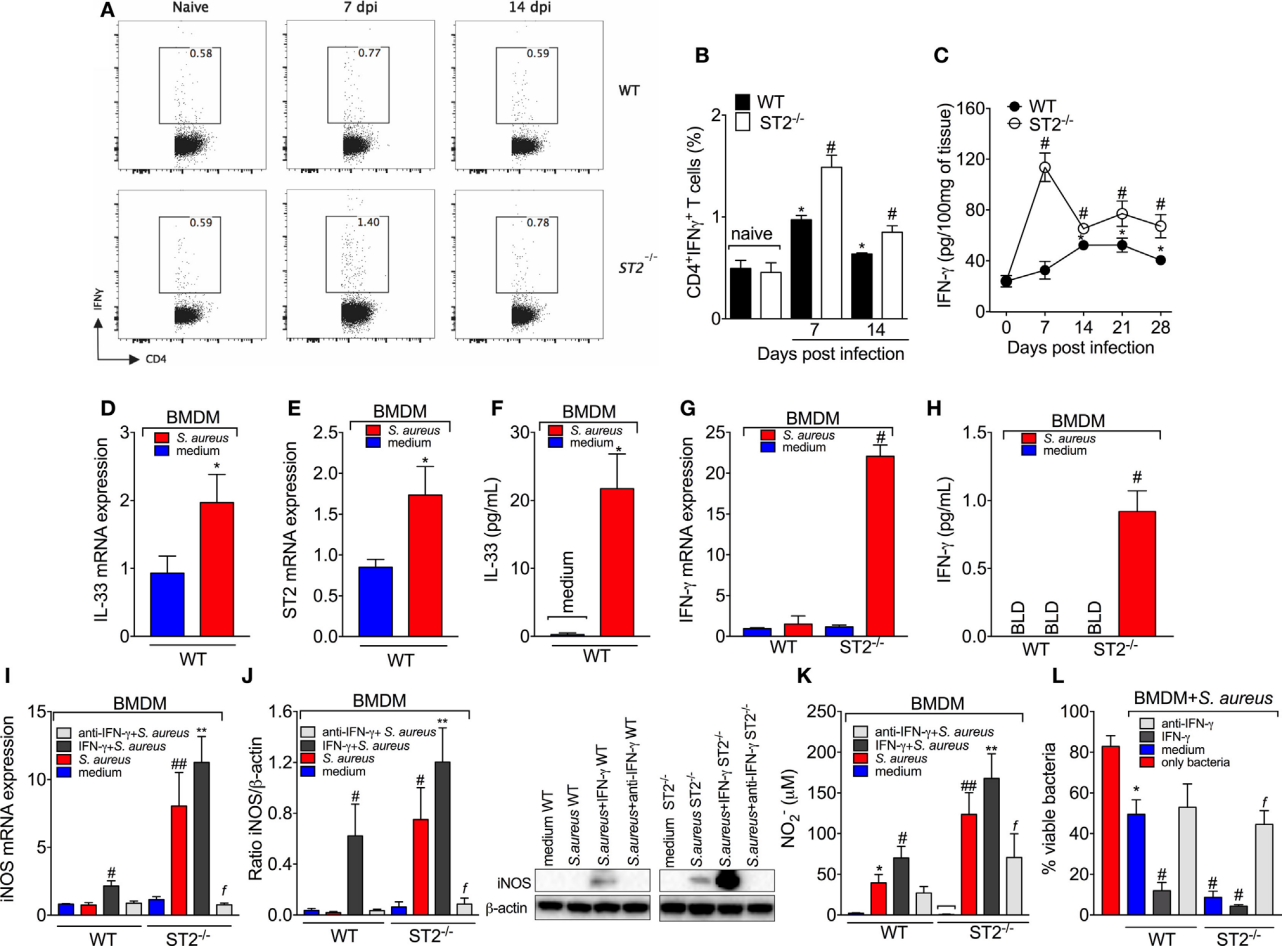
ST2 deficiency enhances type 1-driven immune response against *Staphylococcus aureus* in septic arthritis *S. aureus* or saline was injected in in the femur-tibial joint of wild-type (WT) and ST2^−/−^ mice. **(A)** Representative FACS plots and **(B)** the percentage of interferon-γ (IFN-γ)-producing CD4^+^T cells (CD4^+^IFNγ^+^T cells) from lymph node collected at day 7 and 14 post-infection and evaluated by flow cytometry, and **(C)** IFN-γ concentrations in the knee joints of WT and ST2^−/−^ at 7–28 days post-infection determined by ELISA. bone marrow-derived macrophages (BMDMs) (1 × 10^6^) from naïve WT mice were incubated *in vitro* with *S. aureus* at a multiplicity of infection (MOI) of 3 for 18 h to assess: **(D)** interleukin-33 (IL-33) mRNA and **(E)** ST2 mRNA expression by qPCR, **(F)** IL-33 levels by ELISA. BMDMs (1 × 10^6^) from naïve WT and ST2^−/−^ mice incubated *in vitro* with *S. aureus* at a MOI of 3 for 18 h to assess: **(G)** IFN-γ mRNA expression by qPCR and **(H)** IFN-γ levels by ELISA. BMDMs from naïve WT and ST2^−/−^ mice preincubated 1 h *in vitro* with IFN-γ (100 IU/ml), anti-IFN-γ (10 µg/ml) or medium, followed by incubation with *S. aureus* at a MOI of 3 for 18 h to assess: **(I)** iNOS mRNA expression by qPCR, **(J)** iNOS protein expression by Western Blot, and **(K)** NO production determined as nitrite concentration by Griess reagent in the culture supernatant. **(L)** BMDMs (1 × 10^6^) from naïve WT and ST2^−/−^ mice preincubated 1 h *in vitro* with IFN-γ (100 IU/ml), anti-IFN-γ (10 µg/ml), or medium, followed by incubation with *S. aureus* at a MOI of 3 for 3 h to assess bactericidal capability of BMDMs by *Killing* assay. *N* = 5 per group per *in vivo* experiment. *P < 0.05 vs WT naïve group **(B)** or vs day 0 of infection **(C)**, ^#^*P* < 0.05 *vs* WT mice group **(B,C)**. Representative of two independent experiments. One-way ANOVA followed by Tukey’s test. For *in vitro* experiments: Samples were pooled from 5 mice per group, N 5 wells per group per *in vitro* experiment. *P < 0.05 vs WT medium group **(D–F, K)** or only bacteria group **(L)**. ^#^*P* < 0.05 vs WT *S. aureus* group **(G–I)** or vs WT medium **(L)**, ^##^*P* < 0.05 vs WT IFN-γ + *S. aureus* group and WT *S. aureus* group **(I,K)**, **P < 0.05 vs ST2^−/−^ *S. aureus* group and WT IFN-γ + *S. aureus* group **(I–K)**, ^f^*P* < 0.05 vs ST2^−/−^ *S. aureus* group **(I–L)**. Representative of two independent experiments. One-way ANOVA followed by Tukey’s test.

Proper Th1 response alongside with antibiotic therapy is fundamental to kill *S. aureus* (47, 51). Previous evidence shows that IL-33/ST2 signaling favors the expansion of type 2 cells; however, there is also evidence that IL-33 enhances type 1 immune responses (1, 4). Thus, to further address the mechanism underlying the outcome of ST2 deficiency in septic arthritis, it was next performed a flow cytometry gating CD4^+^IFN-γ^+^T cells and CD4^+^IL-4^+^T cells at the 7th and 14th day post-infection in LNs cells. These time points were chosen based on ST2^−/−^phenotype observed during septic arthritis and on the fact that CFU count in the joint was higher at the 7th and 14th day post-infection. Flow cytometry data show that ST2 expression was essential to the development of a Th2 response in septic arthritis (Figures S2A,B in Supplementary Material). We showed that ST2^−/−^ mice had higher number of gated CD4^+^IFN-γ^+^T cells than WT mice at days 7 and 14 post-infection (Figures 5A,B). In contrast, the *S. aureus* induced an increase of the percentage of gated CD4 + IL-4 + T cells at days 7 and 14 post-infection in WT mice, which was reduced by ST2 deficiency (Figures S2A,B in Supplementary Material). In line with FACS data, WT mice presented reduced amounts of IFN-γ and increased amounts of IL-4 in infected joints, which are opposed results compared to ST2^−/−^ at all time points evaluated (7–28 days post-infection, Figure 5C; Figure S2C in Supplementary Material). This suggests that endogenous IL-33 is essential to driving type 2 response in septic arthritis which are mediated by IL-4. This axis favored the infection progression by counteracting type 1 response. In view of Th1 and Th2 response have long been balancing one another (52) and that there are similar evidences regarding the relationship between Th1 and Th17 (52, 53), we found a lower number of gated IL-17^+^CD4^+^T cells in LNs from ST2^−/−^ mice compared with WT at 7th and 14th day post-infection (Figures S2D,E in Supplementary Material), which lined up with IL-17 amounts in infected joint tissue at all time points evaluated (7–28 days post-infection, Figure S2F in Supplementary Material), raising the possibility that ST2^−/−^ mice use Th1 cells in lieu of Th17 cells to drive the immune response against *S. aureus*-induced septic arthritis. We also found that WT mice presented increased number of CD4^+^ IL-10^+^ T cells in LNs when compared with ST2^−/−^ mice at the 7th day post-infection (Figures S2G,H in Supplementary Material). This high IL-10 production in the beginning of infection in WT mice possibly favored bacterial growth (54). At the 14th day post-infection, there was a decrease switch in the number of CD4^+^ IL-10^+^T^+^ cells observed in WT and ST2^−/−^ mice with an increase in ST2^−/−^ mice (Figures S2G,H in Supplementary Material) and it lined up with the IL-10 amounts in infected joints at all times points evaluated (7–28 days post-infection, Figure S2I in Supplementary Material). Thus, increasing CD4^+^IL-10^+^T^+^ cells and IL-10 production at later time points in the course of septic arthritis at which the infection was already controlled could be a mechanism to reduce joint inflammation in ST2^−/−^ mice.

M1 macrophage polarization can be driven by microbial infection and secondarily drive the T helper (Th1) polarization under influence of IFN-γ. We observed that *S. aureus* induced the mRNA expression of IL-33 (Figure 5D) and ST2 (Figure 5E) as well as IL-33 (Figure 5F) production by BMDM. Therefore, the infectious agent induces IL-33 production, which is in line with an altered response in the absence of IL-33 receptor, ST2. IFN-γ is crucial for NO production and evidence demonstrates that BMDM produce IFN-γ and NO in response to *L. amazonensis* (55). In this sense, we found that *S. aureus*-induced IFN-γ mRNA (Figure 5G) and protein (Figure 5H) only in ST2^−/−^ BMDM, but not in WT BMDM. This result explains why adding IFN-γ to the media could not increase the microbicide activity of ST2^−/−^ BMDM (Figure 4B) and suggests that the effect of ST2 deficiency depends on inducing IFN-γ in BMDM in addition to increasing Th1 cell skewing. Corroborating this conclusion, anti-IFN-γ antibody treatment inhibited the enhanced iNOS mRNA expression (Figure 5I), iNOS levels (Figure 5J), NO_2_^−^ production (Figure 5K), and bacterial killing (Figure 5L) of ST2^−/−^ BMDMs compared with WT BMDMs in response to *S. aureus*. These results confirm that the protection against *S. aureus* conferred by ST2 deficiency depends on inducing IFN-γ production. It is likely that *S. aureus* stimulus induces IL-33 that acting *via* ST2 limits IFN-γ production by BMDM functioning as an endogenous regulator of anti-bacterial mechanisms, and upon ST2 deficiency, IFN-γ can be produced to unleash the full microbicide activity of BMDMs.

### IFN-γ Contributes to the Resolution of Staphylococcal Arthritis

**Figure 6:**
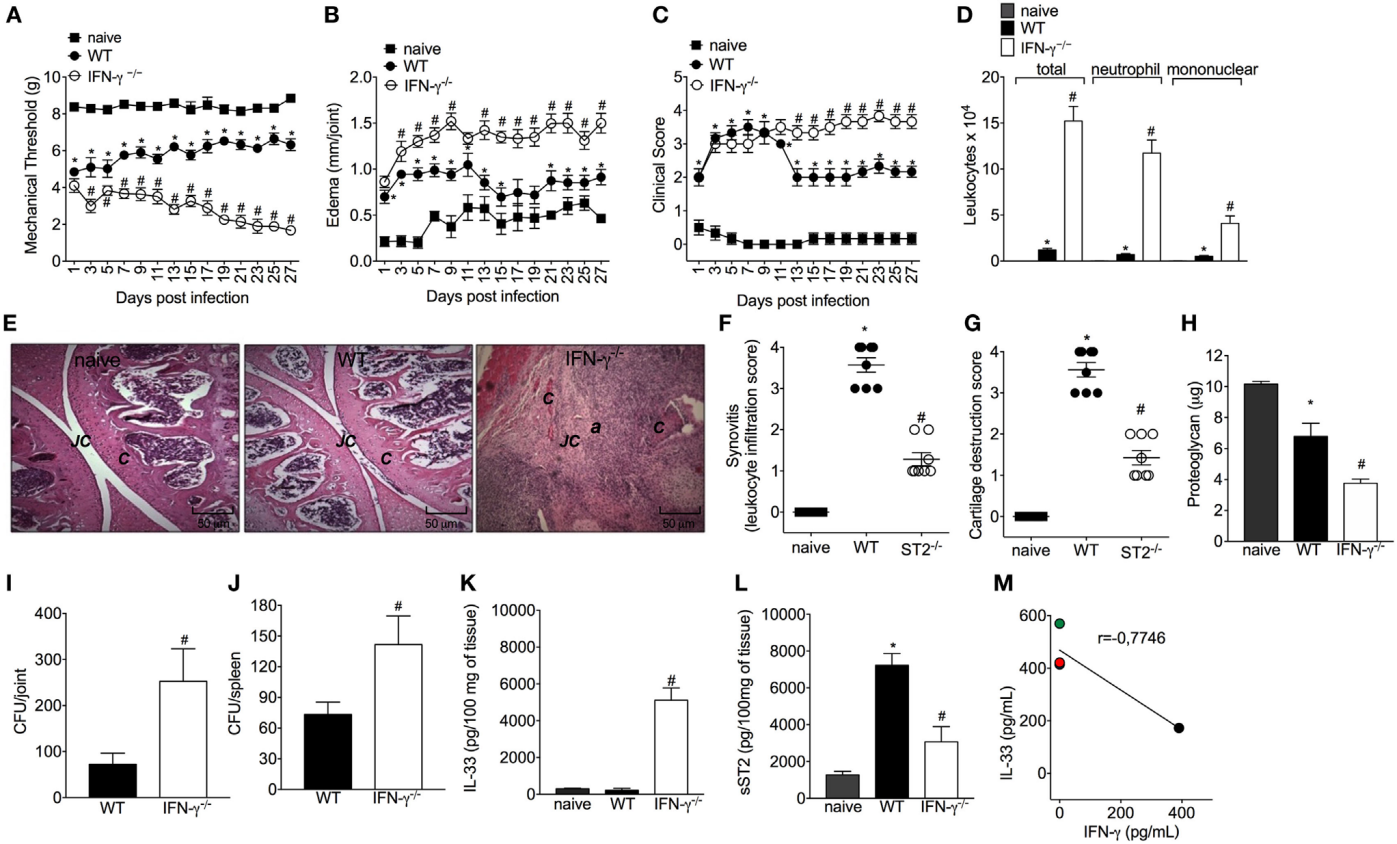
Interferon-γ (IFN-γ) contributes to the resolution of staphylococcal arthritis. *Staphylococcus aureus* or saline was injected in in the femur-tibial joint of wild-type (WT) and IFN-γ^−/−^ mice. **(A)** Mechanical hyperalgesia, **(B)** articular edema, and **(C)** clinical score were evaluated over 27 days post-infection. At 28-day post-infection, knee joint samples were collected at 28-day post-infection to determine: **(D)** leukocyte recruitment to the articular cavity, histopathological analysis of hematoxylin/eosin stained slices: **(E)** representative images of knee joints at 28 post-infection in original magnification ×10. The letter ***a*** indicates a heavily inflamed joint with cartilage destruction and pannus formation, **(F)** synovitis score (intensity: 1–4), and **(G)** cartilage destruction score (intensity: 1–4). At 28-day post-infection, **(H)** proteoglycan content in patellas, **(I)** bacterial counts in knee joint cavity, **(J)** and were determined, and **(K)** interleukin-33 (IL-33) and **(L)** sST2 concentrations in knee joints were determined by ELISA. Correlation analysis of IL-33 and IFN-γ levels determined by ELISA in synovial fluid samples from patients with septic arthritis. For inflammatory parameters and proteoglycan content: *n* = 6 per group per *in vivo* experiment, representative of two independent experiments. *P < 0.05 vs naïve mice group, ^#^*P* < 0.05 vs WT mice group **(A–D,H–L)**. One-way ANOVA followed by Tukey’s test. For histological analysis: *n* = 8 per group per experiment, representative of two independent experiments. *P < 0.05 vs naïve mice group, ^#^*P* < 0.05 vs WT mice group **(E–G)**. Kruskal–Wallis test followed by Dunn’s test. Spearman rank correlation test was used for the assessment of correlation **(M)**. Abbreviations: ***C***, cartilage; ***JC***, joint cavity.

Considering the importance of IFN-γ in the bacterial clearance and that ST2 deficiency enhances IFN-γ^+^CD4^+^T cells skewing and IFN-γ-dependent killing activity of BMDM, it was then investigated whether mice lacking IFN-γ presented impaired response in septic arthritis and whether this was related to IL-33 production. Mice lacking IFN-γ presented increased mechanical hyperalgesia and edema in all disease course (Figures 6A,B, respectively). The differences in clinical features were more prominent from day 13 post-infection as observed by an increase in clinical score in IFN-γ^−/−^ when compared with WT mice (Figure 6C). IFN-γ^−/−^ mice presented increased leukocyte recruitment inflammatory cell infiltrate with predominance of polymorphonuclear leukocytes over mononuclear cells (Figures 6D–F), remarkable cartilage destruction, and architecture as confirmed by proteoglycan content loss and histopathology (Figures 6F–H). The septic arthritis development pattern was thoroughly different comparing WT and IFN-γ^−/−^ mice as observed by the knee joint edema and clinical score (Figures 6B,C). In fact, a lower bacterial killing was observed resulting in higher CFU counts in the joint and spleen of IFN-γ^−/−^ mice compared with WT mice (Figures 6I,J). Ultimately, this profile observed in IFN-γ^−/−^ mice was related to higher IL-33 production, and lower sST2 levels than WT mice (Figures 6K,L). All the parameters were observed up to or in the 28th day post-infection (Figure 6). Importantly, the same dose of *S. aureus* was used in WT C57BL/6 and WT Balb/c mice to induce septic arthritis. Both mouse strains were susceptible to infection; however, there was a clear difference in the disease intensity. The C57BL/6 mouse with pronounced type 1 and type 3 immune responses presented lessened disease severity that allowed observing the disease exacerbation in IFN-γ^−/−^ mouse (Figure 6). The WT Balb/c with a more prominent type 2 profile could not inhibit bacterial growth. The ST2 deficiency enhanced the type 1 immune response resembling the WT C57BL/6 profile, indicate that IFN-γ deficiency impairs the immune response against *S. aureus*-induced septic arthritis similarly to Balb/c mice expressing ST2, which lines up with the data showing that IFN-γ deficiency increased IL-33 production. Thus, enhancing IFN-γ is an essential mechanism to solve septic arthritis and can be achieved by ST2 deficiency. Finally, we quantitated IFN-γ in synovial fluid samples of patients with septic arthritis and compared with the IL-33 levels observed in Figure 1A. An inverse relationship between the levels of IL-33 and IFN-γ was observed (Figure 6M and Figure S3 in Supplementary Material). Making a parallel of the data presented in this manuscript and Figure 6M, we would suggest that with higher levels of IL-33, lower levels of IFN-γ will be observed in the synovial fluid of septic arthritis patients, and changing the balance of this relationship may interfere with the disease outcome.

## Discussion

The present study demonstrates that the IL-33 receptor, ST2, contributes to the development of *S. aureus*-induced septic arthritis. This contribution is drastic in a manner that ST2 deficiency resulted in a better disease outcome. ST2 deficiency increased the Th1 skewing and induced IFN-γ production by BMDM that presented a boosted response with enhanced bacteria killing *via* NO. Septic arthritis presents severe and permanent joint sequelae (29, 31), and the present data suggests that ST2 deficiency ameliorates this infections disease.

The knees are the most common joints that are affected in septic arthritis in humans (27). The diagnosis of septic arthritis depends on isolating the pathogen from aspirated synovial fluid from knee joints (29, 56). We observed that patients have significantly more IL-33 and no detectable concentrations of sST2 in their synovial fluid compared those of the osteoarthritis patients. In the mouse model of septic arthritis, substantial amounts of IL-33 as well as markedly reduced sST2 levels were observed in the knee joint. Thus, the mouse septic arthritis model replicates this IL-33/sST2 balance observed in septic arthritis patients. These clinical and experimental findings are consistent with the notion that the IL-33 levels are tightly regulated by sST2 availability (1, 4, 11, 57), suggesting that during *S. aureus* septic arthritis there is an enhanced availability and, potentially, activity of IL-33.

Infectious disease is the outcome of an intense crosstalk between invading pathogen and host defense armory (58). In septic arthritis, the synovial membrane inflammation leads to tissue destruction and dysfunction, resulting into significant painful condition and morbidity (28, 56, 59). In this context, the effect of ST2 deficiency in staphylococcal arthritis was evident since the first day after bacteria injection, inducing articular hyperalgesia, edema and a focal collection of immune cells that secret proinflammatory mediators such as TNF-α and IL-1β, which in turn contribute to the knee joint lesion, abscess and function loss (28). Indeed, IL-33 can orchestrate the influx of neutrophils and other immune cells subsidizing a dysfunctional joint inflammation in other arthritis models (13–15, 60). Conversely, using ST2 deficient mice, we observed a decrease in articular hyperalgesia, clinical score, and all inflammatory profile of staphylococcal arthritis over 28 days as result of a better control of infection, establishing a relationship between ST2 deficiency and intrinsic factors modulating microbicide mechanisms. The initial phenotype results could represent a reduction of inflammatory response since IL-33 induces hyperalgesia and edema (13, 41). Reduced inflammation would result in diminished killing of bacteria, which would be in line with evidence that in ovalbumin-induced airway inflammation IL-33 does not affect NO production from iNOS (61). However, this was not true in the present experimental condition since ST2 deficiency resulted in a better killing activity and control of staphylococcal arthritis avoiding the infection to become systemic. The ST2 deficiency allowed an enhancement of a Th1 cells activation of neutrophils and macrophages with IFN-γ with enhanced anti-bacterial activity. Unexpectedly, ST2 deficient macrophages produced IFN-γ for an autocrine induction of iNOS expression/NO production and killing activity, thus, suggesting that ST2 deficiency also favors an M1 macrophage phenotype that contributes to protect the host against *S. aureus* infection. Corroborating these data, an essential role for NO and other NO congeners in controlling septic arthritis was demonstrated (28, 62).

Extrapolating to others bacterial infections, IL-33/ST2 axis has apparent pleiotropic functions shaped by the local microenvironment (4, 19, 21, 38, 63, 64). Our findings appear to contradict the reports showing the beneficial role of IL-33 on bacterial infections, including cutaneous *S. aureus* infection (21, 63, 64). One fundamental explanation is that even before the recognition of IL-33, it was demonstrated that ST2 makes a negative-feedback control of IL-1RI and TLR signaling *via* sequestration of MyD88 and Mal through TIR domain (3, 65). Direct activation of TLRs in neutrophils, specifically TLR2 in staphylococcal infections, downregulates the expression of chemokine receptor CXCR2 that keeps their recruitment to infectious foci. Thus, once at infectious focus, neutrophils no longer need to continue migrating, but they must solve the infection by producing microbicide molecules such as NO (19, 66). Further, NO produced from iNOS also downregulates chemokine receptor expression (19, 67). Considering that IL-33 interferes with TLR signaling (19), it is reasonable to suppose that in ST2^−/−^ mice with septic arthritis, TLR2 signaling will be boosted allowing neutrophils and macrophages to produce larger amounts of NO *via* iNOS to eliminate the bacteria. This signaling also links innate and Th1-biased adaptive response (65). Moreover, acute sepsis also presents differences with septic arthritis that contribute to understand the pleiotropic roles of IL-33. Acute sepsis is dependent on innate immune responses, although it triggers immune suppression in the patients that survive (38). On the other hand, septic arthritis is a disease with extremely aggressive acute effects, but also with a chronic temporal profile that allows the development of adaptive immune response as observed here in. The present data supports that ST2 deficiency will allow the development of Th1 adaptive immune response and a better control of infection *via* enhanced IFN-γ production and consequent increase microbicide activity of phagocytes.

NO has a selective enhancing effect on the induction and differentiation of Th1 but not Th2 cells (49, 68, 69). Th1 cells in a feedback loop amplify NO synthesis further *via* activation of M1 macrophages by IFN-γ and suppress IL-4 synthesis, leading to the production of large amounts of NO that is essential for killing pathogens (68). Importantly, a strong Th1 response is desirable for effective host defense against *S. aureus* (63). Conversely, a skewing toward a type 2 immune response is thought to contribute to impaired immune defenses in *S. aureus*-induced septic arthritis (70). Consistent with the inability of WT mice in controlling the infection, these mice showed Th2 profile in septic arthritis with high-IL-4/CD4^+^T cell and low-IFN-γ/CD4^+^T cell counts. In the absence of ST2, the polarization of lymphocytes and type 2 cytokines production was abolished allowing the skewing toward Th1 phenotype. The ST2 deficiency also inhibited Th17 expansion and promoted IL-10 production. These data are in agreement that IFN-γ production might overlap IL-17 release in septic arthritis (71) and that IL-10 was released in order to achieve a state of tissue homeostasis in chronic phase of septic arthritis (72).

Concluding, we find a novel endogenous role for the IL-33 receptor, ST2, in septic arthritis. Abrogating ST2 expression enhances the Th0 polarization toward a Th1 phenotype that *via* IFN-γ induces iNOS-derived NO production by macrophages and neutrophils improving the killing activity of these innate immune cells. Furthermore, ST2 deficient macrophages produce IFN-γ that boosts their bactericide armory. Therefore, ST2 deficiency is beneficial in *S. aureus*-induced septic arthritis.

## Ethics Statement

Animal care and handling procedures were in accordance with the International Association for Study of Pain (IASP) guidelines, and approved by the Ethics Committee of the Londrina State University (OF.CIRC.CEUA, process number 20165/2009).

## Author Contributions

LS-F, JA-F, and WV conceived and designed the experiments. LS-F, ST, VF, DN, and KL performed the experiments. LS-F and FF pathological analysis. RO and PL-J patient’s recruitment, collection of human samples. LS-F, ST, VF, DN, KL, and WV collection of data and analysis. JP, RC, SF, MT, TC, FL, FC, JA-F, and WV contributed reagents/materials/analysis tools. LS-F, VF, and WV writing-original draft. LS-F, RC, TC, FC, JA-F, and WV writing-review and editing. All authors contributed to manuscript revision, read and approved the final version of the manuscript.

## Conflict of Interest Statement

The authors declare that the research was conducted in the absence of any commercial or financial relationships that could be construed as a potential conflict of interest.
