# Supplementary material for: Interleukin-33 Receptor (ST2) Deficiency Improves the Outcome of Staphylococcus aureus-Induced Septic Arthritis
Source: Front Immunol. 2018 May 16;9:962. doi: 10.3389/fimmu.2018.00962 (PMC5968393; doi:10.3389/fimmu.2018.00962)
Supplement: Supplementary file 1 [file presentation_1.PDF]

Supplementary Material

Interleukin-33 receptor (ST2) deficiency improves the outcome of *Staphylococcus aureus*-induced septic arthritis.

Larissa Staurengo-Ferrari[1], Silvia C. Trevelin[2,3], Victor Fattori[1], Daniele C. Nascimento[3], Kalil A. Lima[3], Jacinta S. Pelayo[4], Florêncio Figueiredo[5], Rubia Casagrande[6], Sandra Y. Fukada[7], Mauro M. Teixeira[8], Thiago M. Cunha[3], Foo Y. Liew[9], Rene Oliveira[10], Paulo Louzada-Junior[10], Fernando Q. Cunha[3], José C. Alves-Filho[3], Waldiceu A. Verri, Jr[1, *].

[1]Departamento de Patologia, Centro de Ciências Biológicas, Universidade Estadual de Londrina, Rod. Celso Garcia Cid KM480 PR445, CEP 86057-970, Cx Postal 10011, Londrina, Paraná, Brazil.
[2]King's College London, British Heart Foundation Centre, Cardiovascular Division, London, United Kingdom.
[3]Department of Pharmacology, Ribeirão Preto Medical School, University of São Paulo, Av. Bandeirantes 3900, Ribeirão Preto, São Paulo CEP 14049-900, Brazil.
[4]Departamento de Microbiologia, Centro de Ciências Biológicas, Universidade Estadual de Londrina, Rod. Celso Garcia Cid KM480 PR445, CEP 86057-970, CxPostal 10011, Londrina, Paraná, Brazil.
[5]Laboratory of Pathology, Faculty of Medicine, University of Brasilia, CEP 70910-900, Brasilia, Brazil.
[6]Department of Pharmaceutical Sciences, Healthy Sciences Centre, Londrina State University, Av. Robert Koch, 60, CEP 86038-350 Londrina, Parana, Brazil.
[7] Department of Physics and Chemistry, School of Pharmaceutical Sciences of Ribeirão Preto, University of São Paulo.
[8]Laboratório de Imunofarmacologia, Departamento de Bioquímica e Imunologia, Instituto de Ciencias Biologicas (ICB), Universidade Federal de Minas Gerais, Belo Horizonte, Brazil
[9]Division of Immunology, Infection and Inflammation, University of Glasgow, Glasgow, UK.
[10]Division of Clinical Immunology, School of Medicine of Ribeirao Preto, University of Sao Paulo, Avenida Bandeirantes, 3900, 14049-900- Ribeirao Preto, Sao Paulo, Brazil.

***Author for correspondence:** Prof. Waldiceu A. Verri Jr, PhD. Present address: Departamento de Patologia, Universidade Estadual de Londrina, Rod. Celso Garcia Cid KM480 PR445, CEP 86057-970, Cx Postal 10011, Londrina, Paraná, Brazil. Tel: + 55 43 3371 4979. Fax: + 55 43 3371 4387. E-mails: waverri@uel.br or waldiceujr@yahoo.com.br.

SUPPLEMENTARY DATA

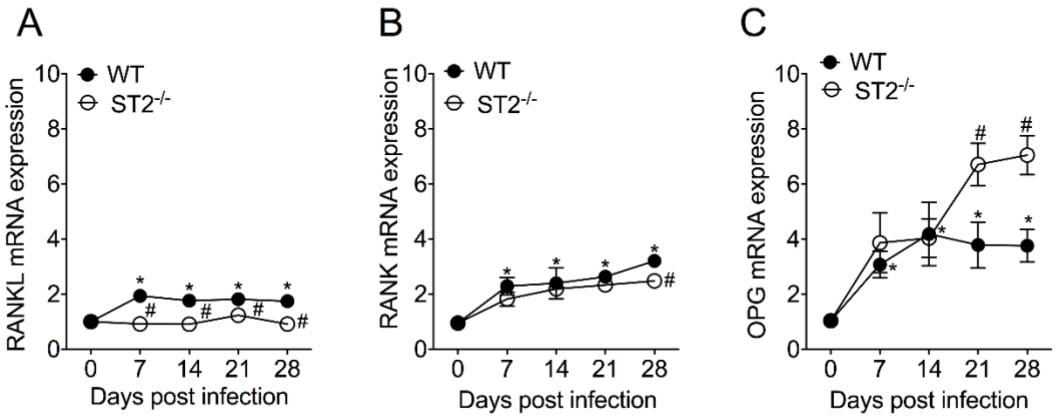

Figure S1. ST2 deficiency affects mRNA expression of the components RANKL/RANK/OPG system. *S. aureus* or saline (day 0) was injected in the fêmur-tibial joint of WT and ST2$^{-/-}$ mice. At indicated points (0 - 28 days post *S. aureus* injection), knee joints were collected and processed to determine the mRNA expression of (**A**) RANKL, (**B**) RANK or (**C**) OPG system by qPCR. N=5 per group per *in vivo* experiment, representative of two independent experiments. *P<0.05 vs day 0 of infection; #P< 0.05 vs WT mice group. Two-tailed unpaired Student's *t*-test.

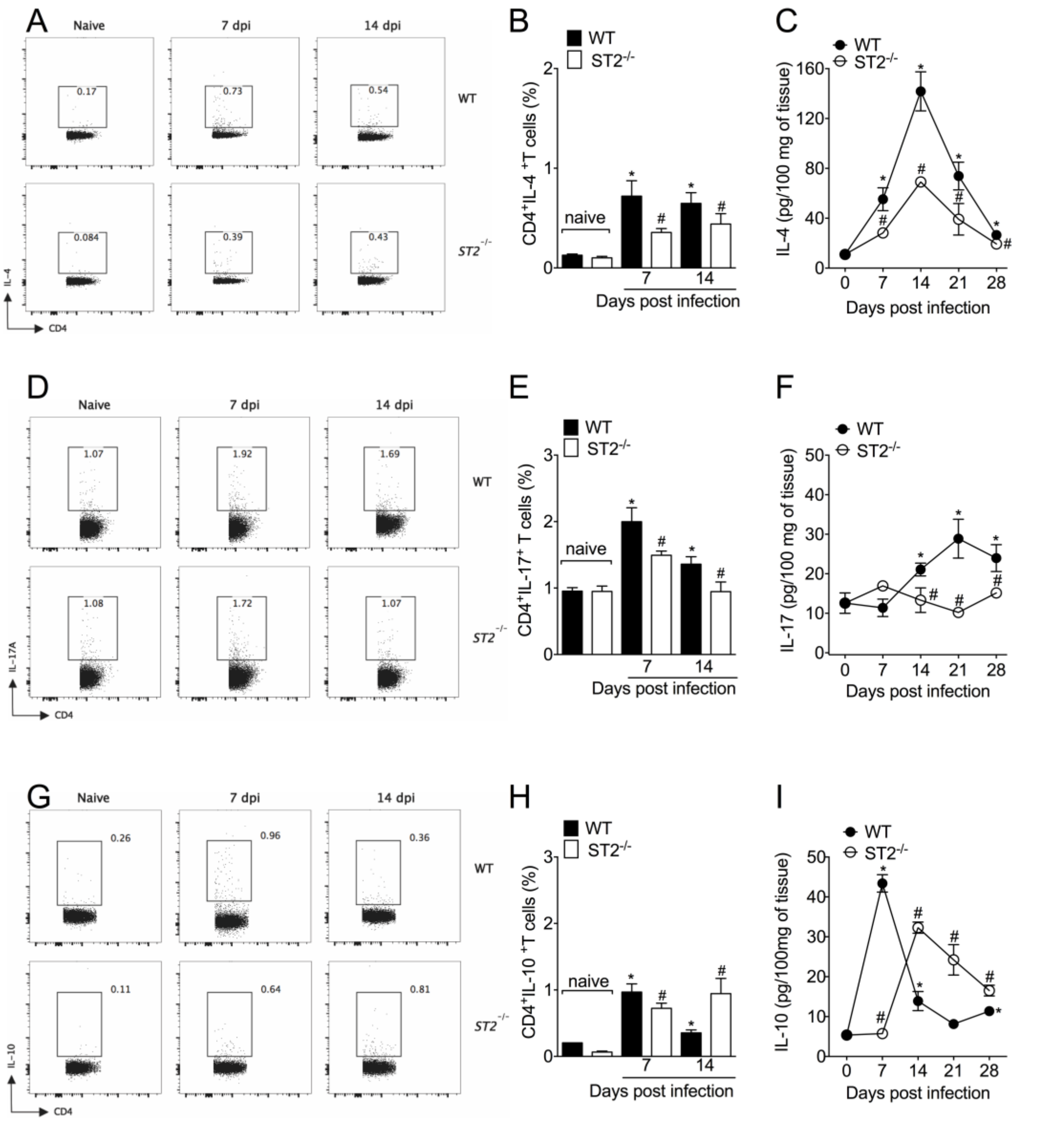

Figure S2. ST2 deficiency affects IL-4, IL-10 and IL-17 producing T cells and cytokine levels in staphylococcal arthritis. *S. aureus* or saline (day 0) was injected in in the femur-tibial joint of WT and ST2$^{-/-}$ mice. (**A**) Representative FACS plots and (**B**) the percentage of IL-4-producing CD4^{+}T (CD4^{+}IL-4^{+}T) cells from LN collected at day 7 and 14 post-infection and evaluated by flow cytometry, and (**C**) IL-4 concentrations in the knee joints of WT and ST2$^{-/-}$ at 7-28 days post- infection determined by ELISA. (**D**) Representative FACS plots and (**E**) the percentage of IL-17-producing CD4^{+}T (CD4^{+}IL-17^{+}T) cells from LN collected at day 7 and 14 post-infection and evaluated by flow cytometry, and (**F**) IL-17 concentrations in the knee joints of WT and ST2$^{-/-}$ at 7-28 days post-infection determined by ELISA. (**G**) Representative FACS plots and (**H**) the percentage of IL-10-producing CD4^{+}T (CD4^{+}IL-10^{+}T) cells from LN collected at day 7 and 14 post-infection and evaluated by flow cytometry, and (**C**) IL-10 concentrations in the knee joints of WT and ST2$^{-/-}$ at 7-28 days post- infection determined by ELISA. N=5 per group per *in vivo* experiment, representative of two independent experiments. *P<0.05 vs WT naïve group; #P<0.05 vs WT mice group. One-way ANOVA followed by Tukey's test.

Supplementary Table 1. Clinical and demographic features of septic arthritis (SA) and osteoarthritis (OA) patients.

**Clinical features**	**SA** (n=5)	**OA** (n=10)
Mean age (years ± SEM)	49.5 ± 8.3	59.6 ± 2.9
Women/Men (%)	3 (60 %)/2 (40%)	5 (50%)/ 5 (50%)
Previous articular disease (%)*	3 (60 %)	0
Immunosuppressive therapy (%)**	3 (60%)	0
Antibiotic therapy (%)	3 (60 %)	0
WBC (± SEM) in SF	44512.5 ± 15.387	675 ± 10.52
Neutrophils (%)	79.8	20.4
Mononuclears (%)	20.2	79.6
RF (± SEM) in SF	10.0 (± 0.2)	9.8 (± 0.1)
S. aureus positive in SF	5	0

*All presented with Juvenile Idiopathic Arthritis, ** two patients with previous corticosteroid infiltration and one in the use of sulfasalazine; RF = Rheumatoid Factor; WBC = White Blood Cells; SF=Synovial Fluid

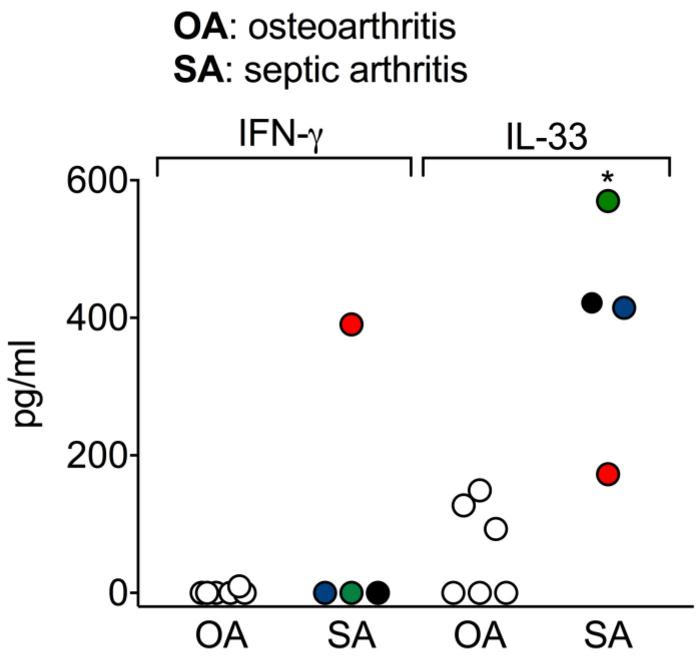

Figure S3. IFN-γ and IL-33 levels in synovial fluids of septic arthritis and osteoarthritis patients. Synovial fluid samples from patients with septic arthritis and osteoarthritis were collected and processed to determine the levels of IFN-γ and sST2 by ELISA. *P<0.05 vs osteoarthritic patients group. Kruskal-Wallis test followed by Dunn's test. Synovial fluid samples of only 4 patients with SA were sufficient for dosing IFN-γ and IL-33.

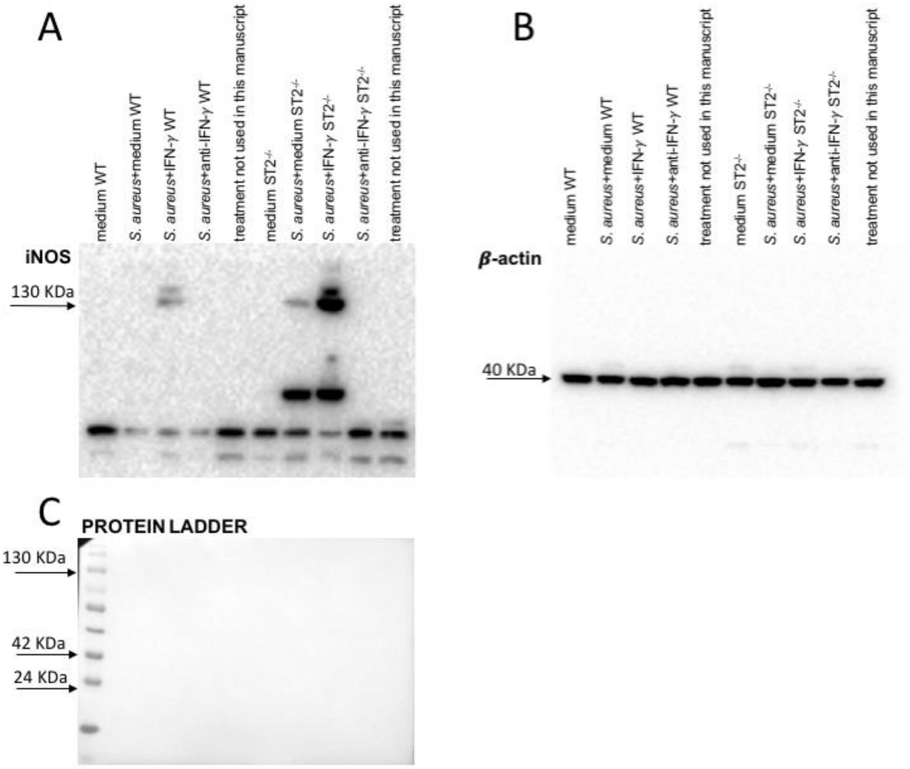

Figure S4. Scan of the original uncropped representative membrane used for western blot analysis. Original Western blots used in Fig. 5H. (**A**) Western blot for iNOS. (**B**) Western blot for β-actin. (**C**) Protein Ladder.
